# Pre-COVID-19 Immunity to Common Cold Human Coronaviruses Induces a Recall-Type IgG Response to SARS-CoV-2 Antigens Without Cross-Neutralisation

**DOI:** 10.3389/fimmu.2022.790334

**Published:** 2022-02-11

**Authors:** Makoto Miyara, Melissa Saichi, Delphine Sterlin, François Anna, Stéphane Marot, Alexis Mathian, Mo Atif, Paul Quentric, Audrey Mohr, Laetitia Claër, Christophe Parizot, Karim Dorgham, Hans Yssel, Jehane Fadlallah, Thibaut Chazal, Julien Haroche, Charles-Edouard Luyt, Julien Mayaux, Alexandra Beurton, Neila Benameur, David Boutolleau, Sonia Burrel, Sophia de Alba, Sasi Mudumba, Rick Hockett, Cary Gunn, Pierre Charneau, Vincent Calvez, Anne-Geneviève Marcelin, Alain Combes, Alexandre Demoule, Zahir Amoura, Guy Gorochov

**Affiliations:** ^1^ Sorbonne Université, Inserm, Centre d’Immunologie et des Maladies Infectieuses (CIMI-Paris), Assistance Publique Hôpitaux de Paris (AP-HP), Hôpital Pitié-Salpêtrière, Paris, France; ^2^ Unit of Antibodies in Therapy and Pathology, Institut Pasteur, Paris, France; ^3^ Unité de Virologie Moléculaire et Vaccinologie, Institut Pasteur, Paris, France; ^4^ Theravectys, Paris, France; ^5^ Sorbonne Université, Inserm, Institut Pierre Louis d’Epidémiologie et de Santé Publique (iPLESP), AP-HP, Hôpital Pitié Salpêtrière, Service de Virologie, Paris, France; ^6^ Service de Médecine Interne 2, Institut E3M, Assistance Publique Hôpitaux de Paris (AP-HP), Hôpital Pitié-Salpêtrière, Paris, France; ^7^ Service de Médecine Intensive Réanimation, Institut de Cardiologie, APHP, Sorbonne-Université, Hôpital Pitié-Salpêtrière, Paris, France; ^8^ Sorbonne Université, INSERM, UMRS 1166-ICAN Institute of Cardiometabolism and Nutrition, Paris, France; ^9^ Service de Médecine Intensive-Réanimation, APHP, Hôpital Pitié-Salpêtrière, Paris, France; ^10^ Sorbonne Université, Inserm UMRS Neurophysiologie Respiratoire Expérimentale et Clinique, Paris, France; ^11^ Service de la pharmacie, Assistance Publique Hôpitaux de Paris (AP-HP), Hôpital Pitié-Salpêtrière, Paris, France; ^12^ Genalyte Inc., San Diego, CA, United States

**Keywords:** COVID-19, humoral immune response, coronaviral infection, IgG, IgA

## Abstract

The capacity of pre-existing immunity to human common coronaviruses (HCoV) to cross-protect against *de novo* COVID-19is yet unknown. In this work, we studied the sera of 175 COVID-19 patients, 76 healthy donors and 3 intravenous immunoglobulins (IVIG) batches. We found that most COVID-19 patients developed anti-SARS-CoV-2 IgG antibodies before IgM. Moreover, the capacity of their IgGs to react to beta-HCoV, was present in the early sera of most patients before the appearance of anti-SARS-CoV-2 IgG. This implied that a recall-type antibody response was generated. In comparison, the patients that mounted an anti-SARS-COV2 IgM response, prior to IgG responses had lower titres of anti-beta-HCoV IgG antibodies. This indicated that pre-existing immunity to beta-HCoV was conducive to the generation of memory type responses to SARS-COV-2. Finally, we also found that pre-COVID-19-era sera and IVIG cross-reacted with SARS-CoV-2 antigens without neutralising SARS-CoV-2 infectivity *in vitro*. Put together, these results indicate that whilst pre-existing immunity to HCoV is responsible for recall-type IgG responses to SARS-CoV-2, it does not lead to cross-protection against COVID-19.

## Introduction

The coronavirus disease 2019 (COVID-19) pandemic has heterogeneously impacted the diverse population groups across the world (1). Whilst some patients are at a higher risk of developing severe disease, others such as children and young adults seem to be better protected. It has thus, been hypothesised that any recent past infections due to the common alpha-coronaviruses (alpha-HCoV); HCoV-NL-63 and -229-E, or beta-HCoV-OC-43 and -HK-U1 could cross-protect against severe acute respiratory syndrome coronavirus 2 (SARS-CoV-2) (2–5). However, till date such cross-neutralising antibody responses have not been reported.

Following a primary infection with SARS-CoV-2, the presence of virus-specific IgM, prior to the appearance of IgG antibodies is to be expected. However, in most COVID-19 patients humoral responses directed toward SARS-CoV-2 are of the IgG isotype instead (6–8). We thus, decided to better delineate this link between predominant IgG or IgM antibody responses to SARS-CoV-2 antigens in COVID-19 patients and their pre-existing immunity to common alpha- and beta-HCoV. We also assessed the IgG reactivity of therapeutic intravenous immunoglobulins (IVIG) manufactured from the plasma samples of healthy donors prior to the COVID-19 outbreak. This was due to their potential capacity to demonstrate pre-existing humoral responses against HCoV infections in the general population (9).

In this work, we show that pre-existing immunity to common HCoV, especially beta-HCoV correlated with a memory-type IgG response directed toward SARS-CoV-2 antigens. This immunity however, did not confer cross-protection against subsequent infection with SARS-CoV-2.

## Results

### SARS-CoV-2 Infection Induces HCoV-Specific Recall Responses

**Figure 1:**
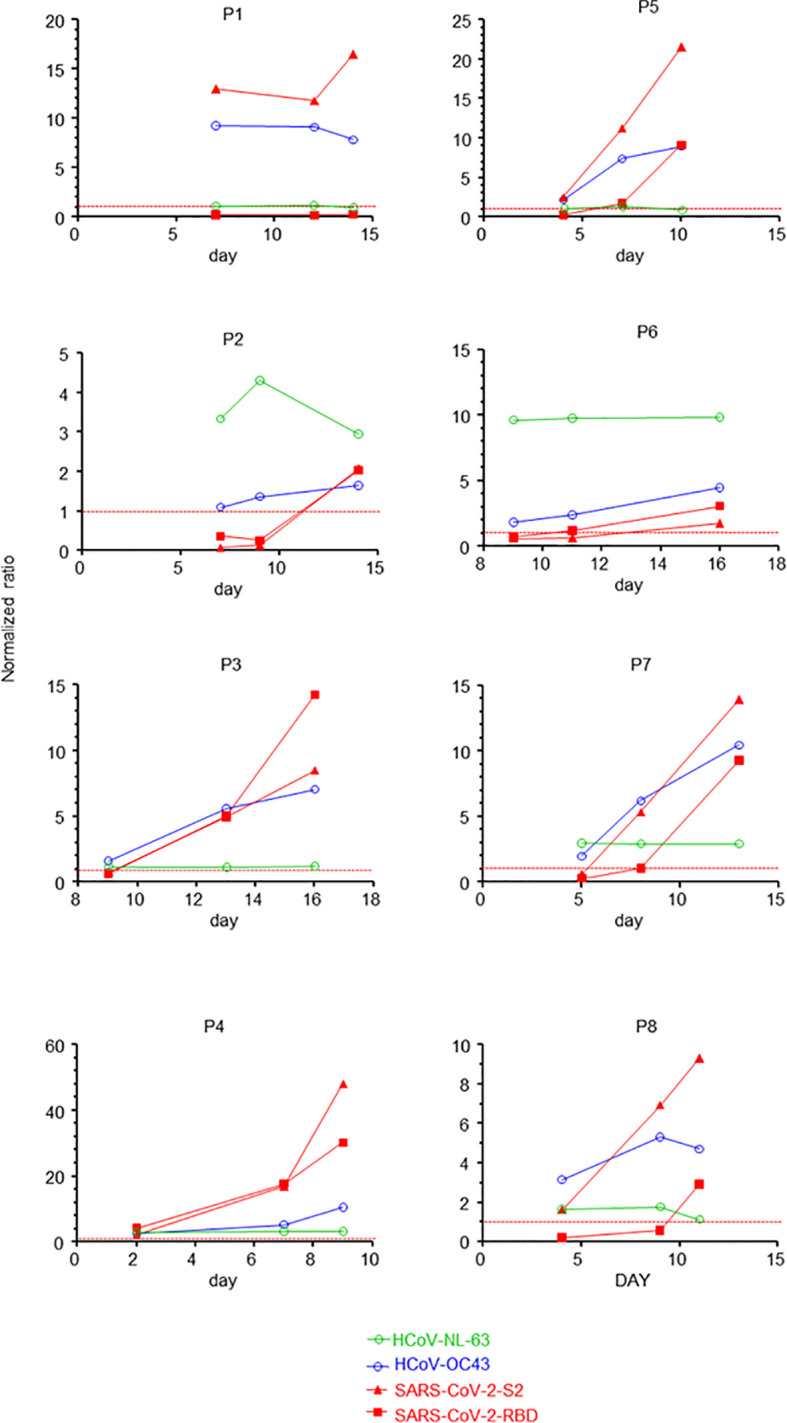
SARS-CoV-2 infection induces HCoV-specific recall responses. Time course of normalised IgG reactivity against SARS-CoV-2 RBD and the S2 domain of the spike protein, alpha-HCoV-NL-63 and beta-HCoV-OC-43 of the sera of eight patients (P1 to P8) with confirmed severe COVID-19. Dotted red lines indicate threshold values for positivity (normalised to 1).

To determine whether humoral cross-reactivity against SARS-CoV-2 and HCoV could be observed during COVID-19, we sequentially analysed the sera of eight severe COVID-19 patients (**Supplementary Table 1**) for their IgG reactivity against SARS-CoV-2 and HCoVs (**Figure 1** and **Supplementary Figure 1**). We found that IgG reactivity against the S2 domain of SARS-CoV-2 Spike protein preceded that against S1 and/or Receptor Binding Domain (RBD). This was also followed by a parallel increase in IgG antibody titres directed towards other SARS-CoV-2 antigens and beta-coronaviruses HCoV-OC-43 and HCoV-HK-U1. However, we did not detect an increase in responses to alpha-HCoV NL-63 or 229-E. The rapid IgG responses to common beta-HCoVs identified were most likely due to cross-reactivity and not as a result of ongoing infections with other HCoVs because their nasopharyngeal RT-PCR was specifically positive for SARS-CoV-2 and negative for all other HCoVs (**Supplementary Tables 1** and **2**). Finally, early sera from all patients showed reactivity against HCoV-OC-43 and HCoV-HK-U1 as they were already present before the appearance of anti-SARS-CoV-2 antibodies (**Figure 1** and **Supplementary Figure 1**).

### Beta-HCoV-Primed Individuals Mount IgG-Dominated Anti-SARS-CoV-2 Responses

**Figure 2:**
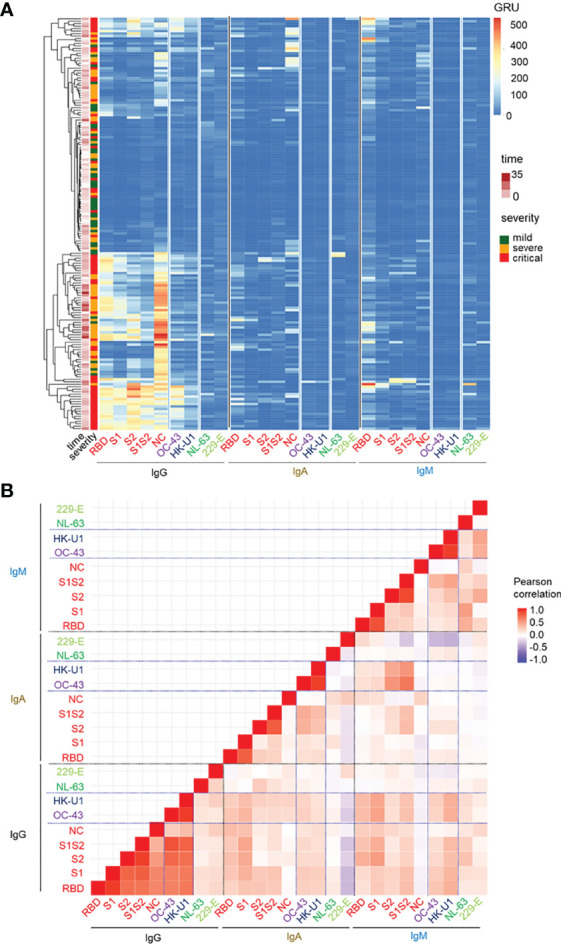
Beta-HCoV-primed individuals mount IgG-dominated anti-SARS-CoV-2 responses. **(A)** Heatmap representation of the IgG, IgA, IgM anti-SARS-CoV-2 virus components (S1, S1S2, RBD, NC) titres (columns) for the entire cohort of patients (rows). Patients were labelled according to their corresponding severity state (moderate, severe, critical) and time point of antibody measurement. **(B)** Pairwise correlation heatmap of the corresponding IgM, IgG and IgA titres in all COVID-19 patients. The Pearson correlation coefficient is colour-coded. The vertical lines separate SARS-CoV-2 and anti-alpha and beta-HCoV Ig-related titres.

We postulated that the appearance of anti-SARS-CoV-2 IgG before IgM could be due to their cross-reactivity against beta-HCoVs. To test this, we retrospectively analysed the titres of anti-SARS-CoV-2 IgM, IgG and IgA, as well as anti-HCoV IgG in the earliest available sera (mean day from symptoms onset: 10.6 days) from 167 patients with COVID-19. Amongst them, 41 had mild COVID-19 that did neither required hospitalisation nor oxygen therapy by nasal cannula. 62 had severe COVID-19 requiring hospitalisation with ward-based oxygen therapy only whereas the remaining 64 patients required admission to an intensive care unit (**Supplementary Table 3**). As demonstrated by the heatmap in **Figure 2A**, the collected sera from all patients confirmed a pattern of high titres of anti-SARS-CoV-2 IgG antibodies, either recognizing the Full Spike, S1, S2 and RBD spike domains or NC in severe and critical COVID-19 patients (10). A strong correlation was also observed between the anti-HCoV-OC-43 and anti-HCoV-HK-U1 IgG responses and the serum levels of anti-SARS-CoV-2 IgG antibodies, in particular those directed against the S2 domain of the SARS-CoV-2 spike protein (r > 0.7, p <0.0001). In contrast, we did not identify any correlation between the IgG responses to SARS-CoV-2 antigens, to HCoV-NL-63 (r=0.05, p-value= 0.051) or HCoV-229-E (r=0.19, p-value = 0.01).

Unlike the IgG responses, there was a poor correlation between IgM and IgA responses to SARS-CoV-2 and those to common alpha- or beta-HCoVs (r<0.27). In comparison, antibody responses to beta-HCoV OC-43 and HK-U1 strongly correlated regardless of the isotype (**Figure 2B** and **Supplementary Figure 2**). Based on these data, we concluded that the IgG responses to SARS-CoV-2 antigens strongly correlated with those to beta-HCoV -OC-43 and -HKU-1, but not common cold alpha-HCoV -NL-63 and -229-E.

It was widely expected that during the COVID-19 outbreak, all patients would develop a primary type antibody response, characterized by the production of anti-SARS-CoV-2 IgM antibodies prior to anti-SARS-CoV-2 IgG and/or IgA antibody seroconversion. However, antibody responses to SARS-CoV-2 in COVID-19 patients were in fact heterogeneous. We observed patients harbouring an early IgG response in the absence of detectable IgM response. In comparison, some had early IgM, but no IgG responses, or even both (**Figure 2A**).

**Figure 3:**
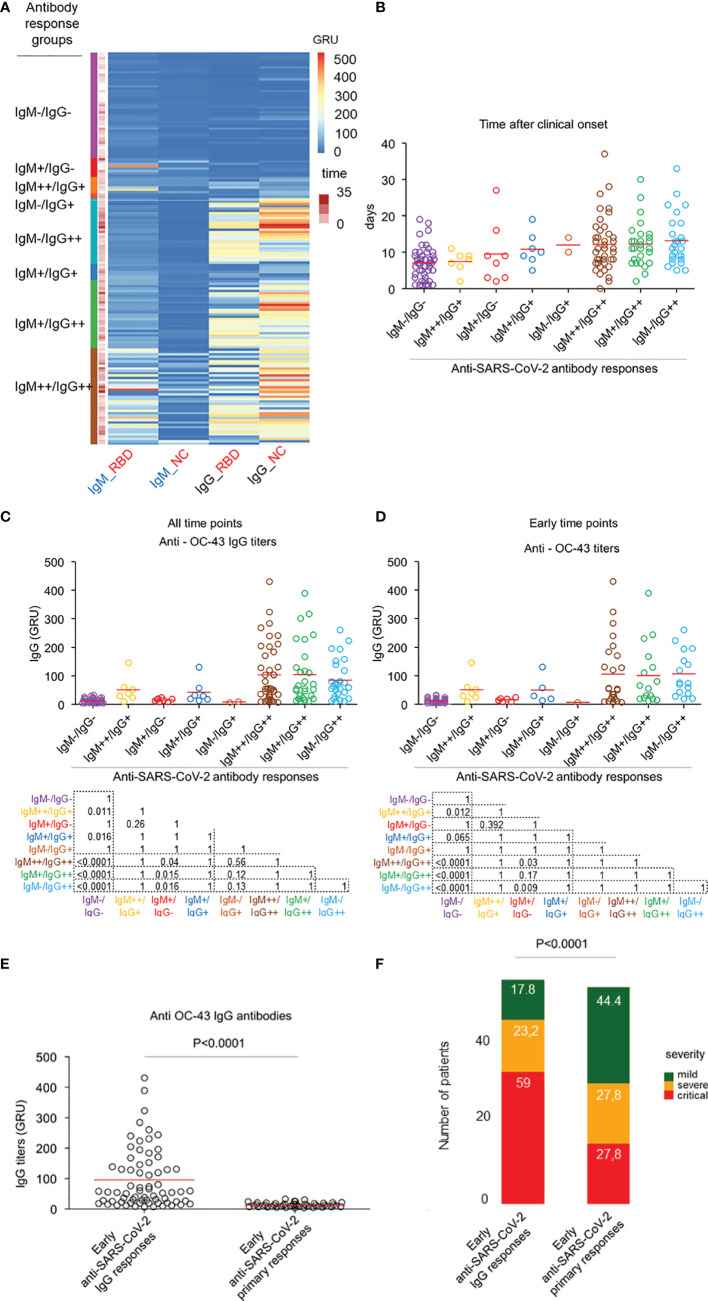
HCoV-induced cross-reactive anti-SARS-CoV-2 antibodies do not protect against COVID-19. **(A)** Heatmap representation of the IgG and IgM titres for NC and RBD SARS-CoV-2 coronaviruses; colors refer to z-score values. Patients were labelled according to their corresponding IgG/IgM subgroup. **(B)** Dot plot representation of the temporal distribution of the 8 identified subgroups of patients; mean time levels are represented in red circles. Dot red line is set at day 12. Sera drawn before or at day 12 are defined as early, while those drawn after are defined as late. **(C)** Dot plot representation of the anti-HCoV-OC-43 IgG antibody responses across all the IgG/IgM subgroups, which are colour-coded. Mean comparison was computed using the Wilcoxon test. **(D)** Dot plot representation of the anti-HCoV-OC-43 (right) IgG antibody across all the IgG/IgM subgroups which are colour-coded with the time points selected within 12 days after clinical onset. Mean comparison using Wilcoxon test was computed between IgM^++^/IgG^++^ and IgM^+^/IgG^-^. **(E)** Dot plot representation of the anti-HCoV-OC-43 IgG responses by early (sera drawn with 12 days after clinical onset) anti-SARS-CoV-2 IgG and early primary response groups. Mean comparison using nonparametric Mann-Whitney *U* test was performed. **(F)** Bar plot representation of the distribution of severity patients across the early IgG responses (n=56) and early primary (n=54) subgroups. All sera were drawn within 12 days after first symptoms. Frequencies of mild, severe and critical cases in each subgroup are indicated in each bar plot. Comparison of the proportions of severity subgroups between the early IgG response and early primary subgroups was made using the Chi-square test.

To analyse the role of such pre-existing immunity to HCoVs in the heterogeneous humoral response to SARS-CoV-2 antigens, we stratified all patients based on the levels of circulating anti-SARS-CoV-2 IgM/IgG (but not IgA) in relation to the timing of blood testing after the clinical onset of disease (**Figure 3A** and **Supplementary Figure 3**). A first subset of patients could be defined as the IgM^+^/IgG^-^ group, characterised by the presence of circulating anti-SARS-CoV-2 IgM, but not IgG antibodies (**Figure 3A**). The titres of anti-HCoV-OC-43 and anti-HCoV-HKU-1 IgG in this group were significantly lower, compared to those in the IgM^-^/IgG^++^ and IgM^+^/IgG^++^ groups (**Figure 3C** and **Supplementary Figure 4A**). Put together, this suggested that patients with a predominant IgM response to SARS-CoV-2 antigens would not have encountered HCoV-OC-43 or HcoV-HKU-1 prior to the outbreak of SARS-CoV-2.

A further group of patients with no detectable serum IgG and IgM antibodies was defined as the IgM^-^/IgG^-^ group. Most of these patients were tested before day 12 after symptoms onset (**Figures 3A, B**). It can be assumed that these patients, due to the lack of an early recall-type IgG response, would eventually develop a primary IgM response. This “non-recall-type response” group also had low titres of anti-HCoV-OC-43 and HCoV-OKU-1 IgG antibodies (**Figure 3C** and **Supplementary Figure 4A**).

Additional groups of patients with weak or strong IgM responses as well as concomitant elevated IgG responses could be defined as IgM^+^/IgG^++^ and IgM^++^/IgG^++^ respectively. It is already known that simultaneous production of IgM and IgG antibodies can be observed during primary responses on one hand and recall antibody responses on the other hand. Therefore, these patient groups could be further subdivided into; (1) early (within 12 days after clinical onset) IgM and IgG response subgroup that corresponds to early recall IgG responses with emerging recall IgM responses and, (2) a late (later than 12 days after clinical onset) IgM and IgG response subgroup that comprises both patients with primary IgM responses seroconverting to IgG responses and those with late recall IgG responses with emerging IgM responses (**Figures 3A, B** and **Supplementary Figure 3**). Moreover, the early IgG and IgM response subgroups had higher titres of anti-beta-HCoVs IgG antibodies than the IgM primary response groups defined above (IgM+/IgG-, IgM-/IgG- “not recall-type response” groups). This further indicated that pre-existing immunity to beta-HCoV was present in COVID-19 patients with IgG recall-type responses (**Figure 3D** and **Supplementary Figure 4B**).

To further examine the role of pre-existing beta-HCoV immunity in the determination of primary or recall-type antibody responses to SARS-CoV-2, we first merged patients with IgM only responses (IgM^+^/IgG^-^ group) or absence of both IgM and IgG (IgM^-^/IgG^-^) within 12 days after clinical onset into a primary response group. Patients with early IgG responses (IgM^++^/IgG^+^, IgM^+^/IgG^+^, IgM^-^/IgG^+^, IgM^+-^/IgG^++^, IgM^+^/IgG^++^, IgM^++^/IgG^++^, groups) within 12 days after clinical onset were merged into a recall-type response group (**see** **Supplementary Figure 3**). We found that anti-HCoV-OC-43 and -HK-U1 IgG antibodies were mostly absent in the primary response group but abundant in the recall-type response group (p<0.0001 for both beta-HCoV) (**Figure 3E** and **Supplementary Figure 4C**). Importantly, the distribution of mild, severe, and critical cases differed significantly (p<0.001) between the two groups with a higher proportion of critical cases in the early recall-type IgG response group. This further indicated that pre-existing immunity to beta-HCoV was not protective against COVID-19 (**Figure 3F**).

Taken together, these results indicate that early anti-SARS-CoV-2 IgG production in COVID-19, reminiscent of a recall-type IgG response was more likely to be found in patients with pre-existing anti-common beta-HCoV IgG. In comparison, primary SARS-CoV-2 infection with dominant IgM early response was more likely to be observed in patients without pre-existing anti-common beta-HCoV IgG antibodies. This pre-existing immunity to common beta-HCoVs, although leading to cross-reactive IgG responses to SARS-CoV-2, failed to prevent the onset of COVID-19 (**Supplementary Table 3**).

### SARS-CoV-2 Antibody Immunity in the Pre-COVID-19 Era

**Figure 4:**
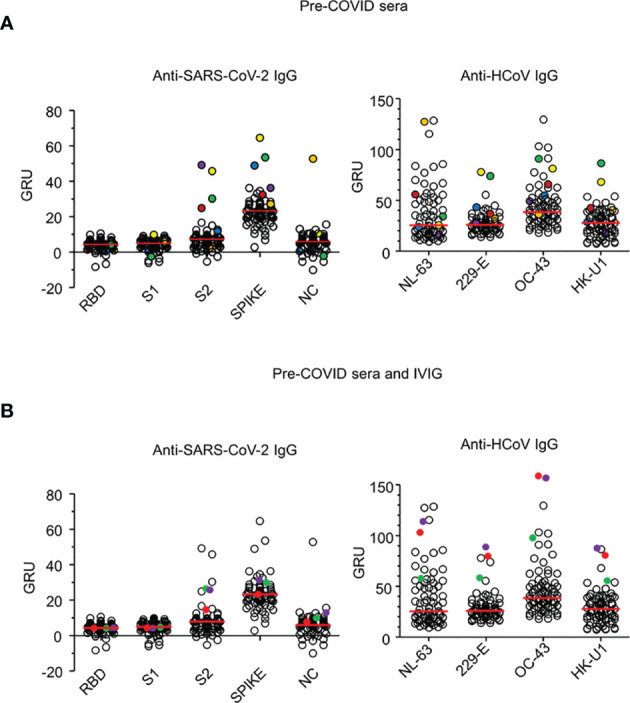
Pre-COVID-19 sera and IVIG reactivity to SARS-CoV-2 and HCoV antigens. **(A)** IgG reactivity of 76 sera drawn from healthy donors in 2015 analysed by phototonic ring immunoassay against SARS-CoV-2 antigens: Receptor binding domain (RBD), S1 domain (S1), S2 domain (S2) of the spike protein, spike and Nucleoprotein (NC), (left) and to the HCoV-OC-43, -229-E and -HK-U1 spike proteins and HCoV-NL-63 nucleoprotein (right). Six sera (C1 to C6) reactive against either SARS-CoV-2 S2, spike or NC are show in circled coloured dots. Reactivity levels are reported in GRU (Genalyte reactive units). Median reactivity is shown with red horizontal lines. **(B)** IgG reactivity of three IVIG batches produced before the outbreak of COVID-19 and the 76 sera, described in **(A)**, against SARS-CoV-2 and HCoV antigens. IVIG batches (IVIG 1 to 3) are shown in plain coloured dots. Antigens are described in **(A)**. Reactivity levels are reported in GRU.

To determine whether the presence of pre-existing immunity to HCoV could also lead to cross-reactivity against SARS-CoV-2 antigens in healthy individuals, we analysed a cohort of 76 healthy French donors (48 males; 28 females; median age of 39 years; age range 19-65, **Supplementary Table 4**) established in 2015. Although we did not detect anti-RBD reactivity in the sera of these individuals, six serological samples (7.9%) were found to be reactive against one or several of the other SARS-COV2 antigens that is, the S2 domain, full-length Spike, and/or Nucleocapsid (NC). These sera also recognized all HCoVs, indicating that pre-COVID-19 cross-reactivity to SARS-CoV-2 antigens was neither specific for a unique family, nor for a particular type of coronavirus (**Figure 4A**).

We also assessed the reactivity of intravenous immunoglobulins (IVIG) manufactured prior to the COVID-19 outbreak. This was done to indirectly identify coronavirus reactivity in a large cohort as therapeutic IVIG consists of IgG isolated from about 10 000 pooled plasma samples of healthy donors. The IgG antibodies of the three different batches of IVIG demonstrated strong reactivity against all HCoVs, as well as detectable reactivity against the SARS-CoV-2 S2 domain and the full-length spike antigen (**Figure 4B**). Collectively, these results indicated that pre-COVID-19 cross-reactivity against SARS-CoV-2 antigens was present in the general population.

### HCoV-Induced Cross-Reactive Anti-SARS-CoV-2 Antibodies Do Not Neutralise SARS-CoV-2 *In Vitro*

In order to experimentally confirm that pre-existing cross-reactivity did not lead to cross-protection against SARS-CoV-2 infection, we assessed the neutralising capacities of the six pre-COVID-19 sera reactive against SARS-CoV-2 antigens and the three IVIG batches through an *in vitro* SARS-CoV-2 neutralisation assay. Whilst full neutralisation of SARS-CoV-2 was observed with sera from COVID-19 patients containing anti-RBD antibodies, pre-COVID-19 serum devoid of detectable anti-RBD antibodies was ineffective. This effect is best illustrated in **Supplementary Figure 5** with sera of a patient being able to neutralise SARS-CoV-2 replication when drawn 17 days after symptom onset but not at day 7. In addition, neither the pre-COVID-19 sera, nor the IVIG batches, were able to neutralise SARS-CoV-2 *in vitro*.

### COVID-19-Era IVIG May Confer Potent Protection Against SARS-CoV-2

**Figure 5:**
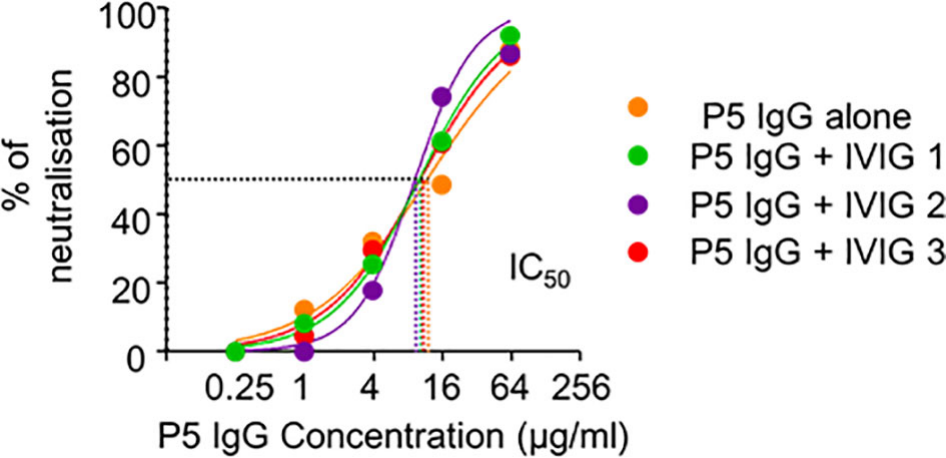
SARS-CoV-2 antibody immunity in pre-COVID-19 era. Neutralisation capacities on the pseudotyped vectors of the IgG isolated from the serum of COVID19 Patient 5 drawn 17 days after symptom onset, diluted with standard diluant (orange) or with each of the three IVIG batches (IVIG-1 in green, IVIG-2 in purple and IVIG-3 in red) with a final IVIG IgG concentration of 12 mg/mL in the neutralisation assay. Half maximal inhibitory concentrations (IC_50_) of patient’s serum alone (11.67 µg/mL) or diluted with IVIG batch 1 (9.94 µg/mL), IVIG2 (9.14 µg/mL) and IVIG3 (10.15 µmg/mL) are shown with dotted lines.

We also assessed the neutralising capacities of IgG isolated from this patient diluted with increasing volumes of IVIG to exclude the interference of a possible inhibitor, inadvertently introduced during the manufacturing process. As shown in **Figure 5**, purified IgG from this patient was able to potently neutralise SARS-CoV-2 *in vitro* even when diluted with IVIG. The half-maximal inhibitory concentrations IC_50_ of the patient’s serum alone (11.67 µg/mL) or diluted with IVIG (IVIG1: 9.94 µg/mL, IVIG2: 9.14 µg/mL, IVIG3: 10.15 µg/mL) were similar. Collectively, these results confirmed that IVIG products manufactured before the COVID-19 outbreak had no neutralising capability.

As shown in **Figure 5**, the addition of neutralising IgG from COVID-19 patients to IVIG preparations could confer this neutralising capability. We had already identified that ~10 µg/mL of COVID-19 patient’s IgG was sufficient to neutralise 50% of the viral cytopathic effect and 64 µg/mL to neutralise all of the viral cytopathic effect in the presence of IVIG (**Figure 5**). Due to our IVIG neutralisation tests being done at 12 mg/mL, we estimated that the presence in serum of only a single SARS-CoV-2-immunized individual out of 1200 donors (0.08%) would be sufficient to confer minimal, but detectable, anti-SARS-CoV-2 activity, while its presence among sera from 187 IVIG donors (0.5%) would confer potent neutralising capacities to IVIG.

## Discussion

In this present study we investigated whether the presence of antibodies against HCoVs in the sera of patients with COVID-19 as well as in healthy donors (isolated prior to the COVID-19 pandemic), could confer protection against SARS-CoV-2 infection. Our results indicated that cross-reactivity not only occurred between SARS-CoV-2 and beta-HCoVs in COVID-19 patients but also with alpha-coronaviruses in healthy individuals. A similar phenomenon against SARS-CoV-2 has also been observed using sera isolated from patients previously infected by SARS-CoV. This was considered to be due to the high degree of homology between the RBD of SARS-CoV and SARS-CoV-2 (5). Interestingly, whilst there are several homologous regions in the S2 domain of SARS-CoV-2 and common alpha- and beta-coronaviruses (**Supplementary Figure 6**), there is no homology between the S1 region, and in particular the RBD region, of SARS-CoV-2 and common alpha- and beta-HCoV (4, 11, 12). Moreover, although antibodies to the RBD of HCoV are commonly detected in most adults, they do not cross-react with the SARS-CoV-2 RBD (11). Put together, our study confirms that pre-COVID-19 immunity to HCoV does not confer cross-protection against SARS-CoV-2 in adults (13). Intriguingly, the opposite effect has been reported in children (14, 15).

Primary responses to novel infectious agents lead initially to the production of IgM then to IgG and IgA. Due to epitope spreading, these antibodies are not necessarily only responsive to the initial pathogen. Therefore, in the context of the COVID-19 outbreak, a primary IgM antibody response against SARS-CoV-2 is to be expected in all patients. However, our results suggest the occurrence of a memory type IgA/IgG response instead in most of the COVID-19 patients. This effect has also been demonstrated by others (7, 16–19). Moreover, this further corroborates our recently reported observation that early anti-SARS-CoV-2 antibody responses are dominated by the presence of IgA and IgG antibodies (20). The latter observation could be explained by the results of the present study showing that immunisation to HCoV, prior to the outbreak of COVID-19, may lead to SARS-CoV-2-specific recall-type IgG and IgA responses. Based on the analogy of epitope spreading, the early IgG reactivity to SARS-CoV-2 antigens in patients that started against the S2 domain, subsequently extended to the S1/RBD domain. (**Figure 1** and **Supplementary Figure 1**). Therefore, one possible mechanism of recall-type responses during COVID-19 might involve epitope spreading starting from epitopes common to HCoV and SARS-CoV-2 toward SARS-CoV-2 RBD. This possibility notwithstanding, variations in the protein sequence between common HCoVs and SARS-CoV-2 RBDs may account for the lack of cross-protection against SARS-CoV-2 by pre-existing anti-HCoV immunity (**Supplementary Figure 5**).

Going further, our findings might be of particular relevance in understanding the the efficacy of the the recently approved SARS-CoV-2 vaccines (21), there are two published reports on large populations receiving the mRNA-based vaccines showing that protection could be achieved as early as 12 days after the first dose (22, 23). This rapid reactivity is usually observed during recall-type immune responses. We would there hypothesise that pre-existing immunity to common benign beta-HCoV might favour an earlier protection against SARS-CoV-2 after a single dose of the vaccine.

Finally, we also demonstrated that IVIG batches produced prior to the outbreak of COVID-19 did not have virus-neutralising capacities. These preparations also did not interfere with the SARS-CoV-2 neutralising capacity of serum IgG. This latter result suggests that IVIG batches manufactured after the COVID-19 outbreak should not exclude donors that have recovered from COVID-19, provided that they do not present potentially deleterious anti-self-reactivity or antibody-dependent SARS-CoV-2 enhancement activity (24). It is nevertheless important to consider that IVIG infusions were reported to be ineffective in non-COVID-19-related SARS (25). Similarly, no such efficacy has yet been demonstrated against COVID-19 using plasma obtained from those that had recovered from the infection (26). Hence, we consider that IVIG batches manufactured during the current pandemic are unlikely to perform a curative role. It remains to be established whether they could instead be used as a prophylactic in early COVID-19 infection.

## Materials and Methods

### Sample Collection

For the healthy donor blood samples, we used previously cryopreserved cells obtained from the French Institute of Blood Donation (EFS, Etablissement Français du Sang, Paris, France). All samples were collected from patients referred to the Pitié-Salpêtrière Hospital. All patient demographic and clinical characteristics are detailed in **Supplementary Table 2**. The provision of samples complied with the guidance from our research ethics committees at the Pitié-Salpêtrière Hospital and Sorbonne Université (CPP - Ile de France-VI and n°2020-CER2020-21). All patients or their relatives gave written informed consent. The three batches of IVIG pharmaceutical products, manufactured before 2019 in France, were obtained from Tegeline-LFB, Clairyg-LFB and CSL Behring. Each batch contained IgG at a concentration of 12 mg/ml.

### Photonic Ring Immunoassay

The presence of serum antibodies specific for the viral antigen was determined using the Maverick SARS-CoV-2 Multi-Antigen Serology Panel (Genalyte Inc. USA). This technology uses an antigen-bound chip to detect the following antibodies for SARS-CoV-2; nucleocapsid, spike S1 RBD, full length spike S1S2, spike S2, and spike S1, as well as the those specific for the common coronavirus HCoV-NL-63; nucleocapsid, HCoV-OC-43, HCoV-229-E and HCoV-HK-U1 spike proteins (27, 28).. It detects and measures changes in resonance when antibodies bind to their respective antigens. All threshold values for positivity were set by the manufacturer. The raw data are shown in **Supplementary Tables 1** **–** **4**.

### Whole Virus Neutralisation Test

The neutralising activity of the sera samples and IVIG was tested with a whole virus replication assay for which a SARS-CoV-2 strain isolated from a SARS-CoV-2 positive patient was used. The virus was isolated by inoculating Vero E6 cells with the patient’s sputum sample in a Biosafety Level-3 (BSL-3) facility. The serum samples were heat-inactivated at 56°C for 30 minutes and following two-fold serial dilutions (from 1:5 to 1:2560), pre-incubated on a 96-well plate with 50 µl of diluted virus (2x10^3^ Fifty percent Tissue Culture Infective Dose 50/mL at 37°C for 60 minutes. Next, 100 µL of the Vero E6 cells suspension (3x10^5^ cells/mL) was added to the mixture and incubated at 37°C with 5% CO_2_ until a microscopic examination was performed on day 4 to measure (or determine) the cytopathic effect (CPE). All neutralising antibody titres were expressed as the highest serum dilution that showed 100% inhibition of CPE. An identical positive serum was added to each experiment as an internal control to assess the reproducibility of the test.

### Purification of IgG From Serum

IgG were isolated from serum samples diluted in 1X-PBS as previously described (29). Briefly, serum samples were loaded onto Protein G/Agarose column (Invivogen) after column equilibration. Chromatography steps were then performed at a flow rate of 0.5 ml/min. Next, 20 column volumes of 1X-PBS were used to wash the column. IgG were then eluted with 5ml of 0.1M glycine (pH 2-3, Sigma-Aldrich) and pH was immediately adjusted to 7.5 with 1M Tris. 1X-PBS buffer exchange was achieved using Amicon^®^ Ultra centrifugal filters (Merck Millipore) through a 100-kD membrane according to the manufacturer’s instructions. Quantification of purified IgG was determined using the NanoVue Plus microvolume spectrophotometers.

### Pseudovirus Production and Permissive Cell Line Generation

Pseudotyped vectors were produced by triple transfection of HEK 293T cells as previously described (30). Briefly, cells were co-transfected with plasmids encoding for lentiviral proteins, a luciferase Firefly reporter, and a plasmid expressing a codon-optimized SARS-CoV-2 spike gene. Pseudotyped vectors were then harvested on day 2 post-transfection. Functional titre (TU) was determined by qPCR after the transduction of a stable HEK 293T-hACE2 cell line. To generate this cell line, HEK 293T cells were transduced at a multiplicity of infection (MOI) 20 with an integrative lentiviral vector expressing the human ACE2 gene under the control of the UBC promoter. Clones were generated by limiting dilution and selected on their permissiveness to SARS-CoV-2 S pseudotyped lentiviral vector transduction.

### Pseudoneutralisation Assay

Firstly, serum dilutions were mixed and co-incubated with 300 TU of the pseudotyped vector at room temperature for 30 minutes. The serum and vector were then diluted in culture medium (DMEM-Glutamax (Gibco), supplemented with 10% foetal calf serum and penicillin/streptomycin (both from Gibco) or with IVIG batches at a 12 mg/mL concentration of the IgG. The samples were then transferred to a tissue culture-treated black 96-well plate (Costar) containing 20x10^3^ HEK 293T-hACE2 cells in suspension. To prepare the suspension, the cell flask was washed with DPBS twice (Gibco) and the cells were individualised with DPBS and supplemented with 0.1% EDTA (Promega) to preserve the hACE2 protein. After 48 hours, the media was removed and bioluminescence was measured using a Luciferase Assay System (Promega) on an EnSpire plate reader (PerkinElmer). The half maximal inhibitory concentrations IC_50_ were determined using Graphpad Prism (version 5).

### Bioinformatics Analyses

All analyses were performed using the R programming language (version 4.2). The heatmaps and correlation plots were generated using “pheatmap” (version 1.0.12). The figures and plots were generated using ggplot (version 3.3.0). For statistical comparisons, we implemented the Wilcoxon test using the “stat_compare_means” function from the ggpubr package. We categorised the patients using reference cut-off values for the NC and RBD, IgG, IgM and IgA titres. The patients were stratified using only the IgM and IgG values given that there was a statistically significant correlation between the IgA and IgG values. The intensity of antibody responses was defined as follows; (-) negative, (+) 1-2 fold above the threshold value and (++) more than 2 fold above the threshold value. When considering the global antibody responses to both RBD and NC (**Figure 4**), the global anti-SARS-CoV-2 antibody response was defined as follows; (-) if both anti-RBD and anti-NC antibody responses were negative, (+) if either anti-RBD or anti-NC or both were positive (but not strongly) and, (++) if at least one antibody response was *strongly* positive. This translated into the following setup:

For each Ig subtype, three categories were set:

*if* NC value AND RBD value < *threshold for* NC/RBD; *then the category is* 0;

else the category is 1.

## Code Availability

All the code used is available on Github: https://github.com/MelissaSaichi/Tfh_GMCSF-DC.

## Data Availability Statement

The original contributions presented in the study are included in the article/**Supplementary Material**. Further inquiries can be directed to the corresponding author.

## Ethics Statement

The studies involving human participants were reviewed and approved by the local ethical committee of the Sorbonne Université (no 2020-CER2020-21). The patients/participants provided their written informed consent to participate in this study.

## Author Contributions

AlM, JF, JH, C-EL, JM, AB, ZA recruited patients. AlM, MM, PQ, ZA collected demographic and clinical data. DS, AuM, LC, KD, CP, PQ, NB prepared the specimens. MM, DS, StM, MA, AuM, FA designed and performed experiments. FA, StM, SB, DB, A-GM, VC, and PC designed, performed and analysed neutralisation assays. SaM, RH, CG designed and provided the Genalyte system reagents. M.S. performed the bioinformatics and biostatistics analysis. MM, MS StM, FA, HY, and DS analysed data. MM, MS, DS, FA prepared the figures. MM, MS, HY, and GG wrote the manuscript draft. MM, ZA, GG designed the study and reviewed the manuscript.

## Funding

MM and GG are supported by the Programme hospitalier de recherche clinique (PHRC: AOR17082,PHRCN 190321), DIM thérapie génique Ile de France and AFPCA (Association Française de la PolyChondrite Atrophiante). DS was supported for this work by a Pasteur/APHP Interface Fellowship. The study was supported by ASPERIM association, Fondation de France, « Tous unis contre le virus » framework Alliance (Fondation de France, AP-HP, Institut Pasteur) in collaboration with the Agence Nationale de la Recherche (ANR Flash COVID19 program), and by the SARS-CoV-2 Program of the Faculty of Medicine from Sorbonne University: ICOViD programs, PI: GG).

## Conflict of Interest

Authors FA and PC were employed by company Theravectys. Authors SA, SM, RH and CG were employed by company Genalyte Inc. MM received consulting fees from Genalyte Inc.

The remaining authors declare that the research was conducted in the absence of any commercial or financial relationships that could be construed as a potential conflict of interest.

## Publisher’s Note

All claims expressed in this article are solely those of the authors and do not necessarily represent those of their affiliated organizations, or those of the publisher, the editors and the reviewers. Any product that may be evaluated in this article, or claim that may be made by its manufacturer, is not guaranteed or endorsed by the publisher.

## Supplementary Material

Supplementary Figure 1 Reactivity of COVID-19 patient sera against SARS-CoV-2 and HCoV antigens. Time course of IgG reactivity against SARS-CoV-2 (left) and to hCoV (right) of sera of eight patients (P1 to P8) with confirmed severe COVID-19. Dotted red lines indicate threshold values for positivity.

Supplementary Figure 2 Pairwise correlation heatmap for IgM, IgG and IgA antibodies titres in all patient groups. Statistical analysis using the Pearson correlation coefficient was carried for each isotype. Results are colour coded.

Supplementary Figure 3 Definition of early anti-SARS-CoV-2 primary and IgG response groups. Patients with antibody responses studied within 12 days after symptoms onset were separated into early anti-SARS-CoV-2 primary response group (absence of IgG, blue boxes), early anti-SARS-CoV-2 IgG response group (detection of IgG, red boxes). Late anti-SARS-CoV-2 IgG response group was defined as detection of IgG (green boxes), after 12 days after symptoms onset.

Supplementary Figure 4
**(A)** Dot plot representation of the anti-HCoV-HK-U1 IgG responses for all (left) and early (sera drawn with 12 days after clinical onset) time points (right) across the, colour-coded, IgG/IgM subgroups, Mean comparison between IgM_-_/IgG^++^ and IgM^+^/IgG^-^ was computed using the Wilcoxon test. **(B)** Dot plot representation of the IgG HCoV-OC-43 responses in the early (sera drawn within 12 days after clinical onset) IgG recall-type response group and early primary response group. Mean comparison was performed using non-parametric Mann Whitney U test.

Supplementary Figure 5 SARS-CoV-2 antibody immunity in pre-COVID-19 era. Neutralisation capacities of pre-COVID sera cross-reactive against SARS-CoV-2 antigens, of IVIG batches and of the sera of Patient 5 at day 7 (P5 d7) and day 17 (P5 d17) after the onset of the symptoms. Neutralisation antibody titres are expressed as the highest serum dilution which shows 100% inhibition of the cytopathic effect of SARS-CoV-2 on Vero E6 cells.

Supplementary Figure 6 Alignment of the protein sequence of the SARS-CoV-2 spike protein with those of alpha and beta-HCoV. SARS-CoV-2 spike protein has been aligned with NL-63, 229E, OC-43 and HK-U1 spike proteins using the BLAST online application (blast.ncbi.nlm.nih.gov). Homologous sequences between SARS-CoV-2 and HCoV are highlighted. Homologous sequences shared by the HCoVs are indicated with identical colours.

Supplementary Table 1–4 Clinical features of healthy donors, patients and antibody titres.
