# Supplementary figures and images for: Pre-COVID-19 Immunity to Common Cold Human Coronaviruses Induces a Recall-Type IgG Response to SARS-CoV-2 Antigens Without Cross-Neutralisation
Source: Front Immunol. 2022 Feb 11;13:790334. doi: 10.3389/fimmu.2022.790334 (PMC8873934; doi:10.3389/fimmu.2022.790334)

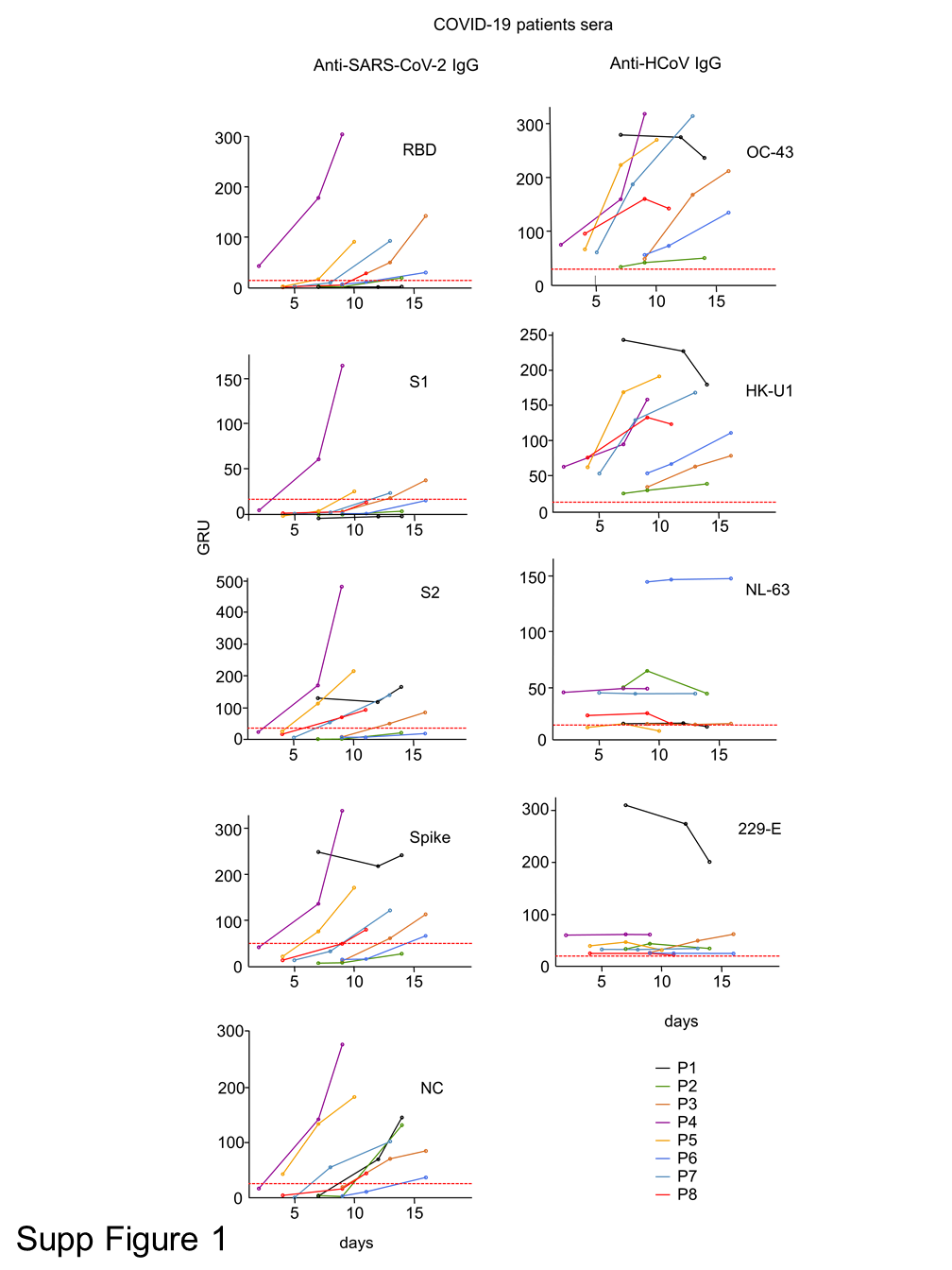

Supplement: Supplementary Figure 1 — Reactivity of COVID-19 patient sera against SARS-CoV-2 and HCoV antigens. Time course of IgG reactivity against SARS-CoV-2 (left) and to hCoV (right) of sera of eight patients (P1 to P8) with confirmed severe COVID-19. Dotted red lines indicate threshold values for positivity. [file Image_1.tif]

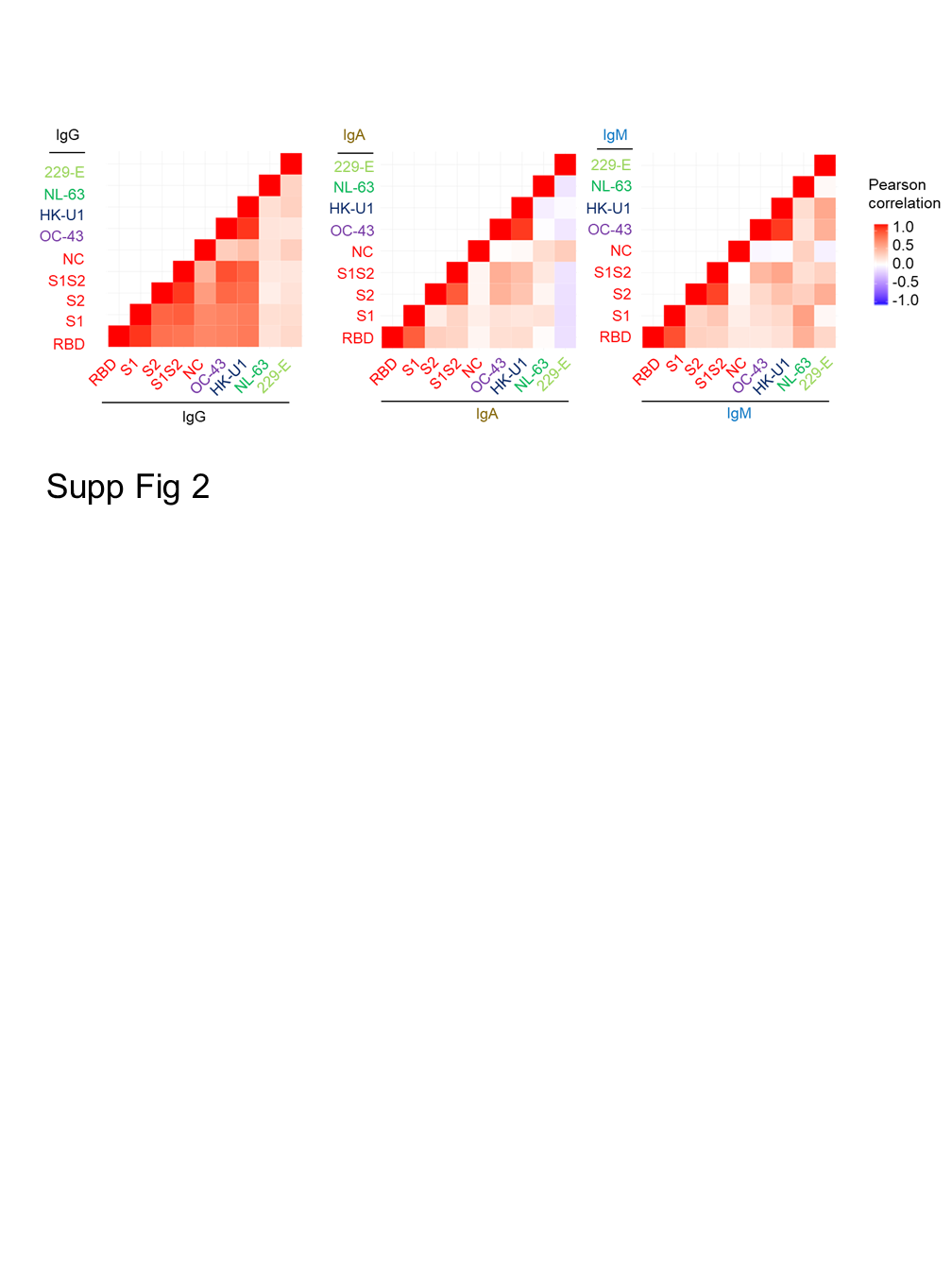

Supplement: Supplementary Figure 2 — Pairwise correlation heatmap for IgM, IgG and IgA antibodies titres in all patient groups. Statistical analysis using the Pearson correlation coefficient was carried for each isotype. Results are colour coded. [file Image_2.tif]

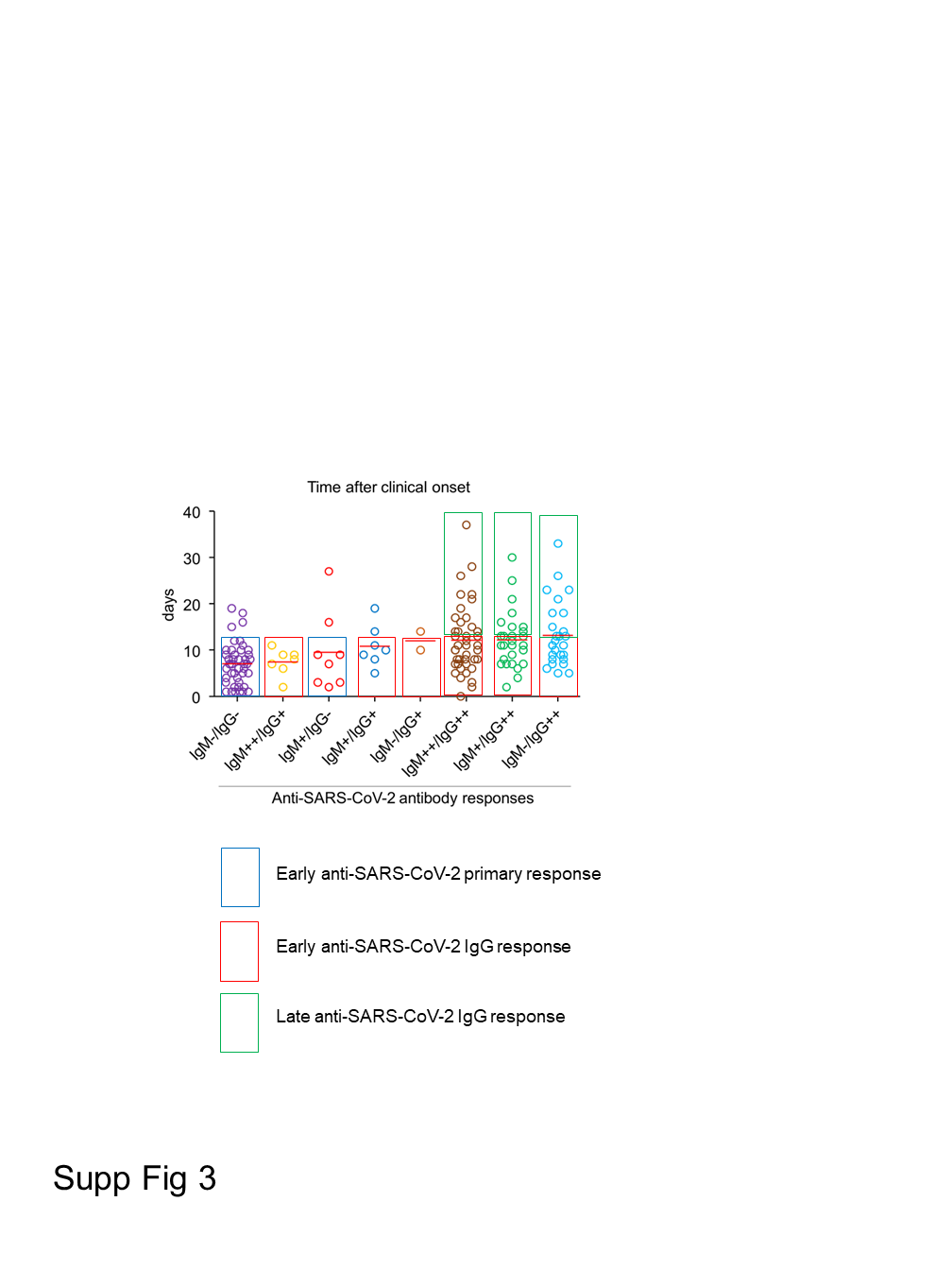

Supplement: Supplementary Figure 3 — Definition of early anti-SARS-CoV-2 primary and IgG response groups. Patients with antibody responses studied within 12 days after symptoms onset were separated into early anti-SARS-CoV-2 primary response group (absence of IgG, blue boxes), early anti-SARS-CoV-2 IgG response group (detection of IgG, red boxes). Late anti-SARS-CoV-2 IgG response group was defined as detection of IgG (green boxes), after 12 days after symptoms onset. [file Image_3.tif]

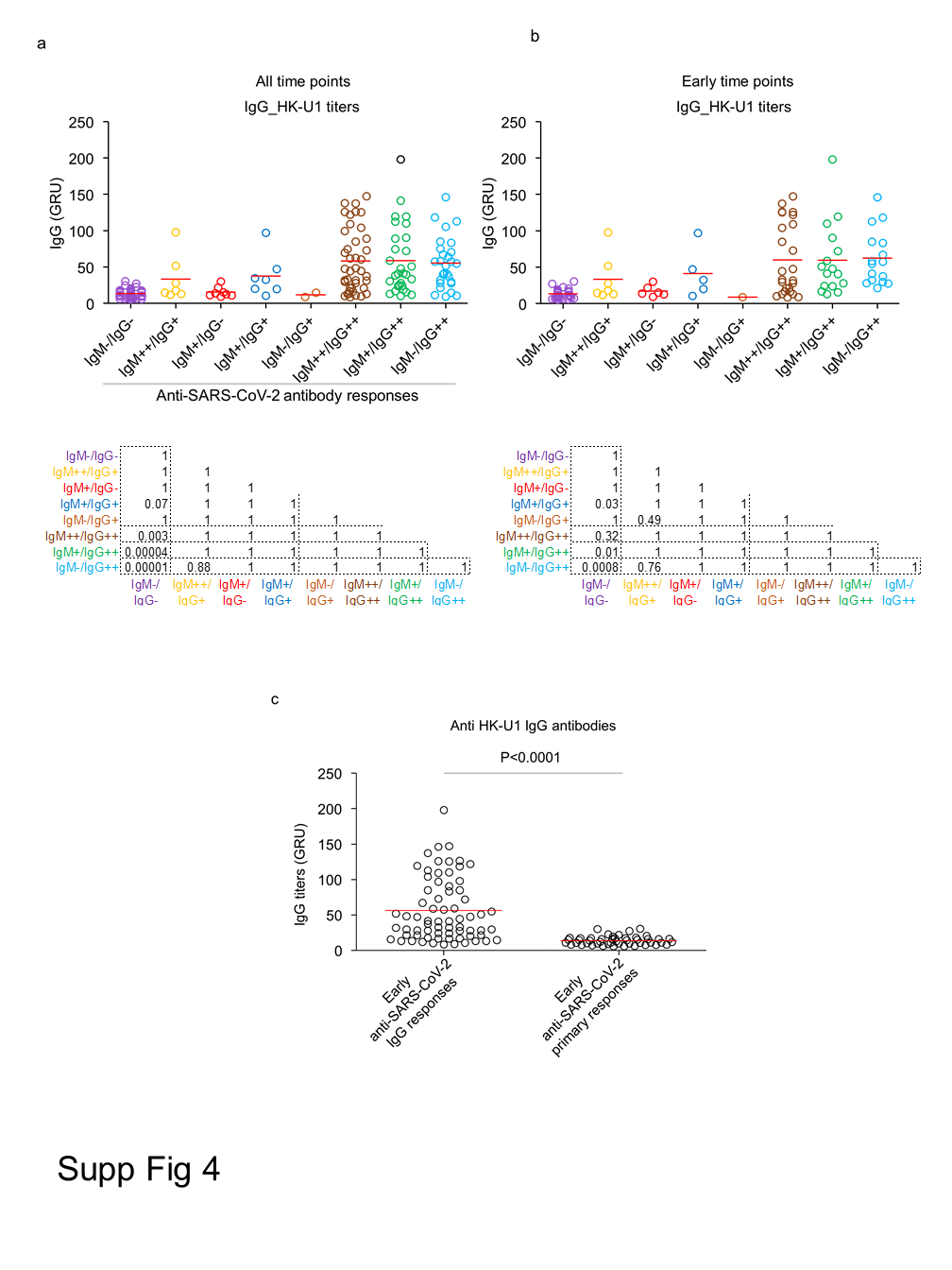

Supplement: Supplementary Figure 4 — (A) Dot plot representation of the anti-HCoV-HK-U1 IgG responses for all (left) and early (sera drawn with 12 days after clinical onset) time points (right) across the, colour-coded, IgG/IgM subgroups, Mean comparison between IgM-/IgG++ and IgM+/IgG- was computed using the Wilcoxon test. (B) Dot plot representation of the IgG HCoV-OC-43 responses in the early (sera drawn within 12 days after clinical onset) IgG recall-type response group and early primary response group. Mean comparison was performed using non-parametric Mann Whitney U test. [file Image_4.tif]

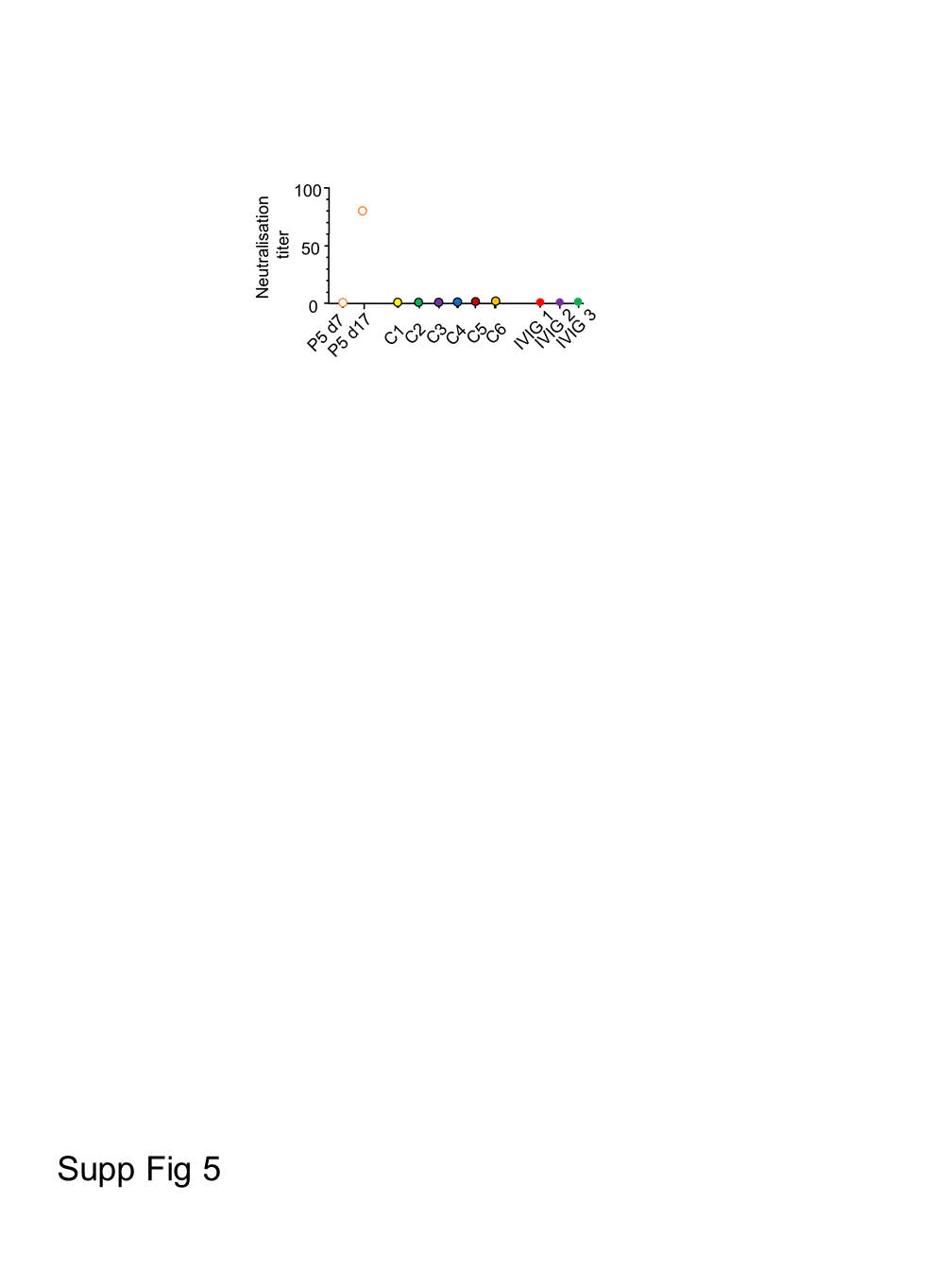

Supplement: Supplementary Figure 5 — SARS-CoV-2 antibody immunity in pre-COVID-19 era. Neutralisation capacities of pre-COVID sera cross-reactive against SARS-CoV-2 antigens, of IVIG batches and of the sera of Patient 5 at day 7 (P5 d7) and day 17 (P5 d17) after the onset of the symptoms. Neutralisation antibody titres are expressed as the highest serum dilution which shows 100% inhibition of the cytopathic effect of SARS-CoV-2 on Vero E6 cells. [file Image_5.tif]

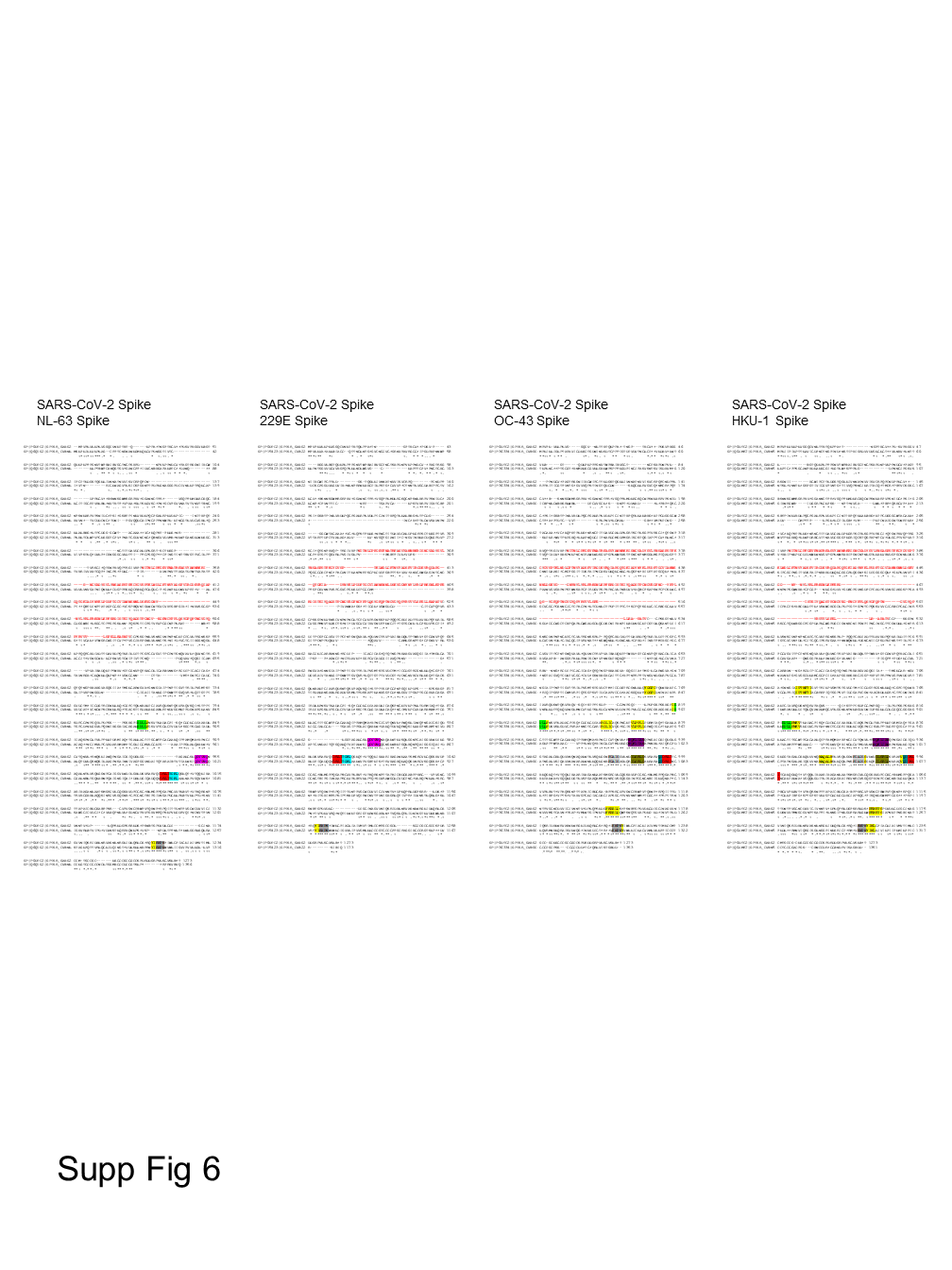

Supplement: Supplementary Figure 6 — Alignment of the protein sequence of the SARS-CoV-2 spike protein with those of alpha and beta-HCoV. SARS-CoV-2 spike protein has been aligned with NL-63, 229E, OC-43 and HK-U1 spike proteins using the BLAST online application (blast.ncbi.nlm.nih.gov). Homologous sequences between SARS-CoV-2 and HCoV are highlighted. Homologous sequences shared by the HCoVs are indicated with identical colours. [file Image_6.tif]
