# Supplementary material for: Pre-COVID-19 Immunity to Common Cold Human Coronaviruses Induces a Recall-Type IgG Response to SARS-CoV-2 Antigens Without Cross-Neutralisation
Source: Front Immunol. 2022 Feb 11;13:790334. doi: 10.3389/fimmu.2022.790334 (PMC8873934; doi:10.3389/fimmu.2022.790334)
Supplement: Supplementary Table 1–4 — Clinical features of healthy donors, patients and antibody titres. [file Table_1.docx]

**Supplementary table 1.** Demographics, baseline characteristics, treatment and outcome of 8 patients with COVID-19

| Patient | Sex | Age (years) | Chronic medical conditions | C-reactive protein (mg/L)  [normal <5] | Naso-pharyngeal  RT-PCR for NL-63, OC-43, 229-E & HK-U1 | pneumonia^‡^ | Chest CT scan: extension of GGO and/or consolidation at admission (%) | Complications | Admission in ICU | Oxygen therapy | Treatments for COVID-19 | Outcomes |
| --- | --- | --- | --- | --- | --- | --- | --- | --- | --- | --- | --- | --- |
| P1 | M | 74 | Hyp, Diab | 79 | Negative | Moderate | 25 | - | No | Nasal cannula (2L/min) | - | Discharged (Day 13) |
| P2 | M | 65 | Hyp, Diab | 77 | Negative | Severe | 25-50 | - | No | Nasal HCM (9L/min) | GCs | Discharged (Day 15) |
| P3 | M | 71 | - | 206 | Negative | Severe | 25-50 | - | Yes | Non-invasive ventilation | - | Discharged (Day 16) |
| P4 | M | 69 | Hyp, Ow,Diab,  COPD | 57 | Negative | Severe | 25-50 | ARDS | Yes | Invasive mechanical ventilation, ECMO | - | Died  (Day 12) |
| P5 | M | 70 | Hyp, Diab | 117 | Negative | Severe | 25-50 | ARDS | Yes | Invasive mechanical ventilation | - | Discharged (Day 42) |
| P6 | M | 83 | Ma Tu | 55 | Negative | Moderate | 25-50 | - | No | Nasal cannula (4L/min) | - | Discharged (Day 8) |
| P7 | M | 46 | Hyp, Diab, Ob | 123 | Negative | Moderate | 25 | - | No | Nasal cannula (4L/min) | - | Discharged (Day 11) |
| P8 | M | 86 | Hyp, Ow, Diab, CVD, COPD | 101 | Negative | Severe | 25-50 | - | No | Nasal cannula (6L/min) | GCs | Discharged (Day 12 ) |

^‡^moderate pneumonia was defined as case showing fever and respiratory symptoms with radiological findings of pneumonia and requiring between 3L/min and 5L/min of nasal cannula oxygen therapy to maintain SpO2 ≥ 96%. Severe pneumonia was defined as requiring above 5L/min of oxygen therapy by nasal cannula to maintain SpO2 ≥ 96%.

ARDS, acute respiratory distress syndrome; CT, computed tomography; COPD, Chronic obstructive pulmonary disease; CVD, chronic vascular disease; Diab, diabetes; ECMO, extracorporeal membrane oxygenation; GGO, ground-glass opacities; GCs, glucocorticoids; HCM, high concentration mask; Hyp Hypertension; ICU, intensive care unit; M, male; Ma Tu, malignant tumor; Ow, overweight; Ob, obese; RT-PCR: Real Time Polymerase Chain Reaction.
