# Supplementary material for: Pre-COVID-19 Immunity to Common Cold Human Coronaviruses Induces a Recall-Type IgG Response to SARS-CoV-2 Antigens Without Cross-Neutralisation
Source: Front Immunol. 2022 Feb 11;13:790334. doi: 10.3389/fimmu.2022.790334 (PMC8873934; doi:10.3389/fimmu.2022.790334)
Supplement: Supplementary file 8 [file Table_2.docx]

| Patient | days post onset of first symptoms | RBD | S1 | S2 | SPIKE | NC | NL-63 | OC-43 | 229-E | HK-U1 |
| --- | --- | --- | --- | --- | --- | --- | --- | --- | --- | --- |
| P1 | 4 | 2.15 | 0.77 | 16.16 | 13.67 | 3.62 | 24.80 | 95.02 | 25.25 | 75.71 |
|  | 9 | 5.85 | 2.29 | 69.40 | 49.41 | 15.37 | 26.87 | 159.99 | 25.15 | 132.81 |
|  | 11 | 29.29 | 12.61 | 93.10 | 80.30 | 43.45 | 17.35 | 141.82 | 21.70 | 123.17 |
| P2 | 9 | 6.32 | 2.71 | 7.54 | 12.02 | 18.62 | 17.25 | 48.07 | 26.83 | 33.54 |
|  | 13 | 50.61 | 17.35 | 49.53 | 61.29 | 69.86 | 16.79 | 167.79 | 49.39 | 62.74 |
|  | 16 | 142.54 | 37.07 | 84.97 | 113.42 | 84.09 | 17.69 | 211.70 | 62.26 | 78.34 |
| 3 | 7 | 3.67 | -0.98 | 0.71 | 7.39 | 2.95 | 50.21 | 33.15 | 33.42 | 24.54 |
|  | 9 | 2.61 | -0.85 | 1.39 | 8.14 | 1.41 | 64.75 | 41.22 | 43.93 | 29.07 |
|  | 14 | 20.40 | 2.94 | 20.83 | 27.83 | 130.96 | 44.53 | 49.63 | 34.56 | 38.28 |
| 4 | 4 | 3.11 | -2.24 | 24.81 | 22.06 | 42.06 | 14.09 | 65.93 | 39.57 | 62.02 |
|  | 7 | 17.47 | 3.15 | 112.83 | 76.08 | 133.05 | 17.34 | 222.95 | 47.07 | 168.72 |
|  | 10 | 91.51 | 24.71 | 215.45 | 171.29 | 182.22 | 10.89 | 269.84 | 31.60 | 191.38 |
| 5 | 7 | 2.48 | -4.94 | 130.07 | 248.46 | 1.87 | 17.41 | 279.08 | 310.24 | 243.33 |
|  | 12 | 1.98 | -2.97 | 117.97 | 217.96 | 68.98 | 17.88 | 274.64 | 274.11 | 227.27 |
|  | 14 | 2.75 | -2.67 | 165.52 | 241.72 | 145.08 | 14.72 | 236.13 | 201.30 | 179.60 |
| 6 | 5 | 3.02 | 0.35 | 5.82 | 13.59 | -0.49 | 45.14 | 60.39 | 32.75 | 52.96 |
|  | 8 | 10.63 | 1.48 | 53.63 | 32.96 | 54.66 | 44.33 | 187.21 | 32.30 | 129.06 |
|  | 13 | 93.17 | 23.01 | 139.50 | 121.81 | 101.42 | 44.53 | 314.09 | 34.71 | 168.22 |
| 7 | 9 | 7.24 | 0.25 | 6.14 | 15.61 | 2.05 | 144.71 | 55.59 | 25.85 | 53.29 |
|  | 11 | 12.07 | 0.35 | 6.58 | 16.24 | 9.77 | 146.87 | 72.33 | 25.15 | 66.58 |
|  | 16 | 30.66 | 14.82 | 17.89 | 66.44 | 35.88 | 147.82 | 134.29 | 24.80 | 110.89 |
| 8 | 2 | 43.61 | 3.91 | 23.10 | 41.69 | 15.70 | 45.55 | 74.31 | 60.30 | 62.40 |
|  | 7 | 177.90 | 60.17 | 170.40 | 136.15 | 141.49 | 49.04 | 159.31 | 61.64 | 94.66 |
|  | 9 | 303.82 | 163.44 | 482.72 | 338.26 | 277.52 | 48.89 | 318.28 | 61.42 | 158.45 |

**Supplementary Table 2** IgG reactivities (measured in GRU) to SARS-CoV-2 and common coronaviruses of patients samples
