# Supplementary material for: Pre-COVID-19 Immunity to Common Cold Human Coronaviruses Induces a Recall-Type IgG Response to SARS-CoV-2 Antigens Without Cross-Neutralisation
Source: Front Immunol. 2022 Feb 11;13:790334. doi: 10.3389/fimmu.2022.790334 (PMC8873934; doi:10.3389/fimmu.2022.790334)
Supplement: Supplementary file 10 [file Table_4.docx]

| **#** | **Gender** | **Age** | **RBD** | **S1** | **S2** | **SPIKE** | **NC** | **NL-63** | **OC-43** | **229-E** | **HK-U1** |
| --- | --- | --- | --- | --- | --- | --- | --- | --- | --- | --- | --- |
| **1** | M | 24 | 4.05 | 4.69 | 2.63 | 21.73 | 4.78 | 84.17 | 21.20 | 34.96 | 21.52 |
| **2** | M | 33 | 4.94 | 0.71 | 3.58 | 11.82 | 1.00 | 19.63 | 53.61 | 25.42 | 23.26 |
| **3** | M | 49 | 5.96 | 2.74 | 3.53 | 15.27 | 13.11 | 49.00 | 102.42 | 41.43 | 39.87 |
| **4** | F | 20 | 4.53 | 5.59 | 2.11 | 22.42 | 5.94 | 24.56 | 44.87 | 32.93 | 37.98 |
| **5** | M | 42 | 5.45 | 5.20 | 5.37 | 20.51 | 7.67 | 67.26 | 35.02 | 28.71 | 10.35 |
| **6** | M | 29 | 0.47 | 7.79 | 12.22 | 27.93 | 7.24 | 127.72 | 47.42 | 25.50 | 11.35 |
| **7** | M | 60 | 6.77 | 4.92 | 4.86 | 25.50 | 6.97 | 53.86 | 53.68 | 24.87 | 17.08 |
| **8** | M | 61 | 1.55 | -4.88 | -2.64 | 9.51 | -5.44 | 50.26 | 32.30 | 26.68 | 26.19 |
| **9 (C5)** | M | 26 | 2.78 | 8.73 | 24.85 | 32.46 | 7.73 | 57.07 | 65.41 | 37.28 | 43.49 |
| **10** | M | 49 | 4.53 | 0.12 | 0.76 | 13.56 | -0.32 | 53.62 | 37.33 | 21.97 | 25.94 |
| **11** | F | 54 | 3.38 | 5.93 | 9.31 | 23.63 | 5.22 | 11.97 | 23.26 | 14.36 | 9.39 |
| **12 (C3)** | M | 49 | 6.27 | 4.05 | 49.24 | 36.27 | 0.38 | 14.10 | 50.60 | 23.82 | 17.89 |
| **13** | M | 28 | 10.53 | 8.64 | 9.75 | 18.70 | 8.65 | 22.37 | 25.32 | 17.34 | 13.13 |
| **14** | M | 27 | 3.18 | 7.75 | 7.82 | 19.34 | 5.66 | 48.55 | 34.35 | 24.49 | 9.08 |
| **15** | F | 49 | 5.74 | 6.51 | 6.72 | 21.23 | 8.95 | 33.64 | 21.30 | 16.75 | 16.22 |
| **16** | M | 57 | 6.46 | 6.29 | 12.69 | 28.46 | 5.47 | 57.43 | 30.86 | 21.52 | 20.61 |
| **17** | F | 38 | 4.62 | 7.14 | 6.49 | 19.98 | 6.33 | 21.05 | 35.83 | 14.46 | 7.58 |
| **18** | F | 27 | 3.39 | 6.21 | 7.70 | 19.27 | 4.70 | 20.12 | 18.19 | 22.68 | 8.31 |
| **19** | M | 49 | 2.11 | -5.49 | -0.76 | 6.84 | -3.11 | 36.56 | 29.87 | 21.91 | 25.26 |
| **20** | F | 22 | 5.09 | 6.34 | 7.78 | 16.75 | 7.15 | 34.03 | 48.08 | 33.96 | 38.73 |
| **21** | M | 52 | 6.10 | 3.19 | 7.30 | 15.71 | 1.12 | 17.99 | 23.56 | 17.63 | 8.13 |
| **22 (C4)** | M | 65 | 2.72 | -0.25 | 9.14 | 48.96 | 0.40 | 20.12 | 49.70 | 42.43 | 31.66 |
| **23** | M | 46 | 7.78 | 0.21 | 4.41 | 12.64 | -0.68 | 34.49 | 18.53 | 16.90 | 8.54 |
| **24** | M | 58 | 2.83 | 7.52 | 9.26 | 28.81 | -10.03 | 30.95 | 24.75 | 17.53 | 13.38 |
| **25** | F | 21 | 5.84 | 7.97 | 7.78 | 22.46 | 9.13 | 21.16 | 91.35 | 21.87 | 48.24 |
| **26** | M | 39 | 0.69 | 8.08 | 15.06 | 33.25 | 6.34 | 115.83 | 38.09 | 25.58 | 33.39 |
| **27** | M | 56 | -6.64 | 3.13 | 7.26 | 16.52 | 2.00 | 25.47 | 37.84 | 16.14 | 18.23 |
| **28** | M | 53 | 5.61 | 6.27 | 9.02 | 34.66 | 9.49 | 23.60 | 43.38 | 20.87 | 32.27 |
| **29** | M | 44 | 5.28 | 5.96 | 3.74 | 20.58 | 6.68 | 55.87 | 26.38 | 28.41 | 7.96 |
| **30** | M | 33 | 5.69 | 2.95 | 9.15 | 25.11 | 6.37 | 14.31 | 23.01 | 17.68 | 18.73 |
| **31** | F | 23 | 4.69 | 3.89 | 5.22 | 26.97 | 10.03 | 12.08 | 57.17 | 40.18 | 39.19 |
| **32** | M | 25 | 5.28 | 6.95 | 12.45 | 27.53 | 10.69 | 15.70 | 32.95 | 22.87 | 21.15 |
| **33** | M | 59 | 5.40 | 3.96 | 8.35 | 19.29 | 2.33 | 35.57 | 45.01 | 23.21 | 25.37 |
| **34** | F | 25 | 3.93 | 6.83 | 7.91 | 18.43 | 5.17 | 14.02 | 62.83 | 21.40 | 21.13 |
| **35** | M | 51 | 3.83 | 5.77 | 9.53 | 22.11 | 6.80 | 40.78 | 29.17 | 28.03 | 17.32 |
| **36** | M | 61 | 5.19 | 2.69 | 7.73 | 19.79 | 1.52 | 14.52 | 25.98 | 25.39 | 26.50 |
| **37** | M | 46 | 1.43 | 6.04 | 9.91 | 36.19 | 3.76 | 66.87 | 35.51 | 22.58 | 41.18 |
| **38** | F | 21 | -0.58 | 5.58 | 3.00 | 18.36 | 5.77 | 25.93 | 62.87 | 43.50 | 40.05 |
| **39** | M | 35 | 0.78 | 5.80 | 6.87 | 23.10 | 6.13 | 16.48 | 28.67 | 16.97 | 21.20 |
| **40** | F | 65 | -1.97 | 4.90 | 7.65 | 26.89 | 10.34 | 22.82 | 25.97 | 28.33 | 8.58 |
| **41** | M | 23 | 5.90 | 2.42 | 4.57 | 20.67 | 4.50 | 17.85 | 62.93 | 33.45 | 25.10 |
| **42** | F | 54 | 6.04 | 1.32 | 0.43 | 14.45 | 0.44 | 47.06 | 77.95 | 33.60 | 40.87 |
| **43** | F | 54 | 6.00 | 5.09 | 5.03 | 19.62 | 8.36 | 40.50 | 21.31 | 26.23 | 11.95 |
| **44** | F | 44 | 4.18 | 3.40 | 5.95 | 21.43 | 4.09 | 77.57 | 129.94 | 25.57 | 53.86 |
| **45** | M | 23 | 4.65 | 5.20 | 1.66 | 23.19 | 7.89 | 28.47 | 42.95 | 21.79 | 33.07 |
| **46** | M | 27 | 2.94 | 5.68 | 1.81 | 27.38 | 6.02 | 13.80 | 41.71 | 23.84 | 26.20 |
| **47** | M | 33 | 6.42 | 7.17 | 9.32 | 25.22 | 7.12 | 19.74 | 50.21 | 30.09 | 40.57 |
| **48** | M | 65 | 8.30 | 9.09 | 11.82 | 25.97 | 15.59 | 16.80 | 44.88 | 31.69 | 33.06 |
| **49** | M | 58 | 5.65 | 7.72 | 7.81 | 20.04 | 9.07 | 64.08 | 24.02 | 17.36 | 28.35 |
| **50** | F | 26 | 5.71 | 5.73 | 5.97 | 22.80 | 7.20 | 70.35 | 40.70 | 45.21 | 35.83 |
| **51** | M | 34 | 3.85 | 4.34 | 6.40 | 17.25 | 5.44 | 10.37 | 34.38 | 26.58 | 28.34 |
| **52** | F | 19 | 2.09 | 7.93 | 8.49 | 24.17 | 5.74 | 15.74 | 38.25 | 32.12 | 21.78 |
| **53** | F | 24 | 5.21 | 7.46 | 4.38 | 20.69 | 7.52 | 20.51 | 31.15 | 18.75 | 20.51 |
| **54** | M | 59 | 8.33 | 2.71 | 4.84 | 14.22 | 6.79 | 16.92 | 27.43 | 19.69 | 25.13 |
| **55 (C1)** | F | 25 | 0.92 | 9.88 | 45.81 | 64.61 | 9.89 | 24.20 | 81.69 | 78.30 | 68.48 |
| **56** | F | 49 | 4.85 | 7.79 | 6.21 | 30.51 | 10.32 | 12.80 | 46.67 | 28.65 | 14.77 |
| **57** | M | 48 | 5.69 | 5.85 | 7.95 | 21.73 | 5.55 | 20.09 | 33.13 | 21.09 | 42.78 |
| **58** | M | 47 | 4.90 | 5.06 | 1.06 | 29.06 | 5.93 | 56.17 | 36.11 | 29.62 | 28.15 |
| **59** | M | 45 | 4.92 | -2.52 | 0.06 | 10.35 | -2.32 | 39.86 | 55.28 | 26.56 | 37.86 |
| **60 (C2)** | F | 28 | 4.25 | -3.43 | 30.32 | 53.56 | -2.13 | 35.65 | 91.68 | 74.28 | 86.88 |
| **61** | F | 27 | 4.18 | 8.19 | 12.21 | 28.45 | 6.19 | 19.25 | 29.63 | 28.90 | 25.76 |
| **62** | F | 31 | -8.40 | 7.62 | 7.93 | 19.67 | 5.14 | 13.64 | 39.20 | 21.72 | 15.02 |
| **63** | F | 34 | 0.89 | 5.64 | 5.43 | 23.48 | 5.53 | 10.97 | 66.21 | 32.27 | 41.56 |
| **64** | M | 22 | 2.82 | 9.34 | 16.64 | 26.54 | 7.47 | 86.12 | 44.80 | 25.17 | 32.99 |
| **65** | F | 33 | 6.43 | 5.85 | 5.54 | 24.21 | 10.62 | 83.50 | 103.57 | 25.73 | 34.00 |
| **66** | F | 33 | 5.67 | -6.02 | -5.34 | 2.72 | -5.43 | 9.72 | 44.19 | 23.89 | 28.95 |
| **67** | M | 46 | 6.80 | 6.86 | 6.90 | 20.53 | 6.82 | 50.71 | 44.49 | 21.06 | 20.85 |
| **68** | F | 24 | 5.91 | 4.41 | 4.49 | 23.02 | 8.20 | 27.51 | 74.06 | 27.27 | 34.52 |
| **69 (C6)** | F | 19 | 7.16 | 6.28 | 2.35 | 24.87 | 52.77 | 128.94 | 35.41 | 30.34 | 40.17 |
| **70** | M | 44 | 4.58 | 7.36 | 6.43 | 23.29 | 6.77 | 34.36 | 34.80 | 23.59 | 35.64 |
| **71** | F | 29 | 5.57 | 9.17 | 9.78 | 20.63 | 6.57 | 22.79 | 34.14 | 27.74 | 19.99 |
| **72** | M | 39 | 6.68 | 4.65 | 6.10 | 24.13 | 4.24 | 19.24 | 51.30 | 36.99 | 54.26 |
| **73** | M | 23 | 4.97 | 6.06 | 9.97 | 19.69 | 4.98 | 22.45 | 36.61 | 19.98 | 24.28 |
| **74** | M | 61 | 9.81 | 6.43 | 3.64 | 27.23 | 12.60 | 17.32 | 49.51 | 38.43 | 34.18 |
| **75** | M | 46 | 3.04 | 5.53 | 1.98 | 22.75 | 6.63 | 16.81 | 49.04 | 27.40 | 33.11 |
| **76** | M | 54 | 4.99 | 3.48 | 5.20 | 21.42 | 3.81 | 46.62 | 53.12 | 55.79 | 47.56 |
|  |  |  |  |  |  |  |  |  |  |  |  |
|  | **IVIG1** |  | 5.64 | 3.47 | 14.44 | 22.99 | 7.69 | 103.69 | 171.17 | 79.29 | 72.62 |
|  | **IVIG2** |  | 6.29 | 3.23 | 26.32 | 35.43 | 9.36 | 113.00 | 158.52 | 88.30 | 87.36 |
|  | **IVIG3** |  | 6.12 | 4.07 | 27.96 | 38.76 | 10.06 | 59.89 | 95.70 | 57.81 | 53.34 |
|  |  |  |  |  |  |  |  |  |  |  |  |

**Supplementary table 4**

Age, gender and IgG reactivities (measured in GRU) to SARS-CoV-2 and common coronaviruses of 76 healthy donors and IgG reactivities of IVIG batches
